# Supplementary material for: Detection of Fused Genes in Eukaryotic Genomes using Gene deFuser: Analysis of the Tetrahymena thermophila genome
Source: BMC Bioinformatics. 2011 Jul 11;12:279. doi: 10.1186/1471-2105-12-279 (PMC3143110; doi:10.1186/1471-2105-12-279)
Supplement: Additional file 1 — Results of Gene deFuser for the Tetrahymena thermophila genome. This zip file contains the raw results of the analysis of the Tetrahymena genome using Gene deFuser. To view the contents, unzip the file and open the Final_Tet.html file in the resulting folder. [file 1471-2105-12-279-S1.ZIP › Results/14.m00425.html]

Gene deFuser -- Results of Job Final\_Tet

 


Gene deFuser

| Home | Retrieve Results | References | Help |
| --- | --- | --- | --- |

Back to Main Results of Job Final\_Tet

# Query Name: 14.m00425

Candidate fusion gene

## Query Sequence:

MKPIRSSSQKSLANSVNSNRASECIQVCIRLRPLLRPQEDEEAWDINKKTQQIYSLIDQKSSLVTNNEITENMSLKDRKRLIEAGNNSIFFTFDNMEGPEVRTQTIYNKMGKHIINSVLEGYHGSILCYGQTTSGKTFTMLGQPENPGILPCSIRDMFNLIQKQANEEFNVWVSYMEIYNENINDLLAPQQQNLKIIEDSKWGTIVQGLKMQRVWNFEQAIVLMNYGEEHRKYRETSIHEHSSRSHTVFKIYLESFSKDLNSGYKKTRYSCLNLVDLAGSERLSEIDFNPNTLGETANCINKLAEGKKQHIPYRDSKLTRILTQALGGNSFTSIICCVSPAVCNFNQTLSTLRFATRAKIIQNKPIINEIGDDISDNQSVSFNEYKKLEENLGQQLKENQTLSQQNIQLKNEISLLQNKMADMSFELNELMNSITLERKKSEALEQDSKRQKITSGNDLLQQSNQEPASINHQDKLQNSLLKNINNEQTFLENAINLVIPMIKEALQLKTQDISHEMEYFYVEKGIKQIVKQICQEEKQFLPIKSFVETLSTLNSISQQNWENKDFQKKGSQAANEGLDKQVQRKQNASNQEEENDEVLQSGRILIQKIEEKQLFRNIQYSFMDIINQLELKESMNEQQIQIILFSLKNQYDQLQKQYDDEYEKYRNELEFFYRRKISVIKNQQISGYNSISLLQEITQDHESKLTILRHIYEQEKNHLEQAYIATIKQIEDCIHRNSYGSIPYNNQEVYKLSNGNLKEAGYFRNYDESQTENSLQYDSSYASSINQSGMSKAGDNSLHRKDINSQKFLQANKQQQALFKTPKFDQFIINQNNSTRSNEPNRIQNELESQITLQKPQYNGIIYIWGNGKDGRLGIEGEKPFKFPQIIPNIQACQVSLGYHHSACISNQGQILTWGRGNRGQLGHSSFENCLLPTPVNFFSKMSIVSVGCGWQHTLALSNDGRVFSWGFGEDGQLGLGDYQSQNSPQFINSLEGYFVTQINAGHSHSSVITSEGIFMMFGCNIDHRLMNKTNQNQFLPCITELEFLRQEYGSDYNAVKASLGVNHSAVITKNGNLYTSGLGNEGQLGSFISEDTPNSLMIQKNSNEMSEYSKFEEEQQENQDQFDRICYFNKVNFFNKKNKAIQVSCGDSFTLVINEQSQVYSFGKGSYYRLGHLDIQTNQNVYEPKLIESLKNFKITSVSTGCRHAVAITNDGKVYTWGFNFYDQLGLGDSEKDYQQPTKVTKLSGLNVKQSSCGYFHTSVLVV

### Significant Ortholog Group Hits and their Scores:

| N terminus | | C terminus | |
| --- | --- | --- | --- |
| [Z] KOG0246 Kinesin-like protein | 40 | [S] KOG1426 FOG: RCC1 domain | 46.4875115431005 |
| [Z] KOG0243 Kinesin-like protein | 39.5570329688403 | [O] KOG0941 E3 ubiquitin protein ligase | 41.4860721670754 |
| [Z] KOG0239 Kinesin (KAR3 subfamily) | 38.8888888888889 | [S] KOG0783 Uncharacterized conserved protein, contains ankyrin and BTB/POZ domains | 26.6159180996187 |
| [Z] KOG0247 Kinesin-like protein | 38.2477581835029 | [S] KOG1427 Uncharacterized conserved protein, contains RCC1 domain | 21.8112646628976 |
| [Z] KOG0242 Kinesin-like protein | 37.4848080015496 | [T] KOG1428 Inhibitor of type V adenylyl cyclases/Neuronal presynaptic protein Highwire/PAM/RPM-1 | 5.24894422419381 |
| [Z] KOG0240 Kinesin (SMY1 subfamily) | 36.6666666666667 |  |  |
| [Z] KOG0245 Kinesin-like protein | 36.3636363636364 |  |  |
| [Z] KOG0241 Kinesin-like protein | 34.2857142857143 |  |  |
| [Z] KOG0244 Kinesin-like protein | 33.7807241485648 |  |  |
| [Z] KOG4280 Kinesin-like protein | 33.6649590255584 |  |  |

#### Graphs (click to enlarge):

|  |  |
| --- | --- |
| BLAST of Query Sequence | Location of Ortholog Group Hits |
|  |  |

Contact: Andre Cavalcanti\_\_\_\_\_Last Modified September 14, 2010
